# AGBT meeting report

**DOI:** 10.1186/s13059-018-1447-8

**Published:** 2018-05-21

**Authors:** Ami S. Bhatt, Christina Curtis

**Affiliations:** Departments of Medicine and Genetics, Stanford University School of Medicine, Stanford, CA 94305 USA

**Keywords:** Applied genomics, de novo genome assembly, Genome regulation, Single cell profiling, Structural variation

## Abstract

The Annual Advances in Genome Biology and Technology (AGBT) General Meeting was held in Orlando, Florida, USA, on the 12–15 February 2018. Professors Ami S. Bhatt and Christina Curtis from Stanford University, USA, report advances and applications in the field that were discussed at the meeting.

## Introduction

The Advances in Genome Biology and Technology (AGBT) meeting has become a staple of the genome biology conference diet and is unmissable for those seeking to digest the ever-quickening advances in genome technology and its applications while enjoying the Florida sun. This year’s meeting covered the themes of emerging single-cell and spatially resolved technologies and the application of genomics to characterize development, health and disease, evolution and ecology. Here we report on several of these themes, noting that owing to space restrictions there are several omissions.

## Mapping organismal development using single-cell sequencing and lineage-tracing approaches

Jay Shendure (University of Washington and Howard Hughes Medical Institute, USA) described new methods for generating global atlases of development, with approaches ranging from single-cell RNA and ATAC sequencing to lineage tracing. Published single-cell RNA sequencing (RNA-seq) of *Caenorhabditis elegans*, resulting in 50× shotgun cellular coverage of the organism, was described, as was single-cell ATAC-seq of embryogenesis, gastrulation, and germ layer formation in the fly. This work in flies, which was subsequently published, outlined the identification of regulatory elements that underlie the major germ layers. In a third (unpublished) study, single-cell ATAC-seq of 13 adult mouse tissues (100,000 cells in total) was described. The resulting data were used to intersect cell type-specific regulatory elements with genome-wide association studies (GWAS) to identify cell types that are functionally implicated in common diseases. Finally, combination approaches were outlined that leverage GESTALT (genome editing of synthetic target arrays for lineage tracing), a lineage-tracing approach combined with single-cell RNA-seq, developed in collaboration with Alex Schier’s group at Harvard University, USA. In summary, substantial progress seems to be possible using a combination of now widespread technical approaches to more completely describe development in higher model organisms.

## A cell hashing approach to facilitate multiplexed single-cell sequencing

Marlon Stoeckius (NY Genome Center, USA) reported on the recently published cellular indexing of transcriptomes and epitopes by sequencing (CITE-seq) method. CITE-seq uses oligonucleotide-tagged antibodies to simultaneously interrogate cellular protein and transcriptome measurements. He further detailed a modification of this approach to enable single-cell multiplexing and doublet detection based on cell ‘hashing’, described in a preprint from the same laboratories. This method enables the pooling of diverse samples (including those that are genetically related and not suitable for genetic multiplexing) by exploiting barcoded antibodies against ubiquitously expressed cell surface proteins, such that the tags can be sequenced in concert with single-cell transcriptomes. The utility of this approach was demonstrated by pooling multiple human peripheral blood mononuclear cell (PBMC) samples in a single 10× Chromium sequencing run, which resulted in a significant reduction in per-cell cost for library generation and minimized batch effects and doublets. Cell hashing has the potential for broad application in other single-cell multiplexing designs and diverse tissues and species, as the reagents for hashing can be expanded to target protein–protein interactions and nuclei.

## Improving contiguity of de novo genomes: genomics and conservation

Beth Shapiro (University of California, Santa Cruz, USA) described the opportunities and technical challenges of using genomic information to inform ecological conservation efforts. Professor Shapiro explored how genomic approaches can inform estimates of population diversity in land animals. Experts have hypothesized that human land use has resulted in dramatic population isolation and inbreeding in the mountain lion *Puma concolor*. To test this hypothesis, the Shapiro group first sequenced and assembled a chromosome-scale de novo genome from a single mountain lion from the Santa Cruz mountains in California, USA. They then generated high-coverage resequencing data from mountain lions across North America and Brazil. Contiguity of standard genome assemblies is generally low, and integration with high-throughput chromosome conformation capture (Hi-C) scaffolding data, which improved contiguity, allowed for more accurate estimations of heterozygosity. In a presentation from our own group (Bhatt, Stanford University, USA), we demonstrated that read cloud-sequencing approaches, such as the 10× genomics linked-read library preparation method, can be paired with customized genome assembly to perform de novo assembly of microbial genomes from complex human and marine microbiomes.

## Detecting copy number and structural variation in archival tumor tissues and single cells

Copy number variation (CNV) and structural variation (SV) are broad-scale genomic alterations that underlie many human diseases, including cancer. High-throughput and sensitive detection of these alterations has been difficult compared with the identification of single nucleotide variants (SNVs) and small insertions and deletions (indels), even with advances in sequencing technology. Several groups described methods that leverage chromosomal conformation and single-cell sequencing to overcome some of the challenges in SV and CNV analysis and to yield high-resolution views of cellular heterogeneity.

Helio Costa (Stanford University, USA) tackled a long-standing problem of identifying SVs in formalin-fixed, paraffin-embedded (FFPE) tissue samples. SVs are commonly found in cancers and can have both diagnostic and predictive utility. However, they are difficult to detect in archival FFPE tissue specimens partly owing to the considerable fragmentation of DNA that occurs during sample processing. The team, which included researchers from Stanford University and Dovetail Genomics, USA, developed a method named Fix-C to overcome this challenge. This method leverages the fact that the first step of FFPE sample preparation—formalin fixation—preserves chromatin organization due to crosslinking. High-molecular-weight DNA was extracted from tumor samples and in many cases exhibited nucleosome-length phasing, suggesting that a modified Hi-C approach would enable the recovery of the geospatial proximity of regions of the genome that have undergone rearrangement. The method was tested on 15 solid tumor specimens and was found to have good concordance with the current clinical gold-standard approach (fluorescence in situ hybridization) and with emerging RNA-sequencing applications. In addition to being able to detect previously confirmed rearrangements, Fix-C also identified several previously undescribed rearrangements and enabled the detection of topologically associated domains.

10× Genomics, a company based in Pleasanton, California, USA, revealed newly developed methods for ATAC-seq and CNV detection in single cells. The CNV detection approach leverages the 10× Genomics single-cell DNA sequencing platform and is a multi-step process: first, the pre-treatment of cells to generate accessible DNA, which is followed by DNA partitioning and single-genome molecular barcoding, and finally fragment pooling and standard Illumina library preparation. This method can be used for primary cells, cells lines, and tissue-dissociated cells in addition to nuclei extracted from flash-frozen tissues. Using this technology, sub-megabase CNVs were detectable in single cells from a breast cancer cell line. Although the method is currently intended for human genomics, there was considerable enthusiasm for the potential adaptation of this and related methods for applications in the metagenomics space.

Nicholas Navin (MD Anderson Cancer Center, USA) reported on a single-cell copy number analysis of 10 patients with breast cancer who were synchronously diagnosed with high-grade ductal carcinoma in situ (DCIS) and invasive ductal carcinoma (IDC), as described in a recent publication. Using laser-catapulting to isolate individual cells and to record their spatial location in an approach that the team named topographical single cell sequencing (TSCS), Navin described tissue maps of copy number variation at single-cell resolution and matched exome sequencing. Four patients were found to exhibit monoclonal DCIS and IDC lesions, whereas the remaining six patients harbored many clones that intermingled and spread across the tissue, similar to reports of subclone mixing in colorectal tumors. In all patients, the same somatic alterations (SNVs and CNVs) were present in the in situ ductal and invasive tumor regions, suggesting that these events arose prior to invasion, which is compatible with a model in which one or more clones escape the ducts and migrate to adjacent tissue to establish the invasive lesion.

## Spatially resolved transcriptomic and proteomic profiling

Joseph Beecham presented NanoString Technologies’ (Seattle, Washington, USA) Digital Spatial Profiling platform that enables spatially resolved multiplexed analysis of protein and RNA expression in FFPE tissues. Their imaging-based method aims to overcome the limitations of standard approaches that have limited dynamic range and multiplexing capacity. Specifically, they build on their nCounter® barcoding strategy to digitally characterize protein and mRNA in multiplex (currently 30 but theoretically up to 800) mRNA probes in cell populations with a dynamic range > 10^5^. The protein assay involves staining of the tissue with antibodies conjugated to photocleavable oligonucleotide tags that are released from user-selected discrete tissue regions via focused (through objective) UV exposure. The cleaved tags are then aspirated using a microcapillary, quantitated using a standard nCounter® assay, and mapped back onto the tissue location. Beecham highlighted the utility of this approach to characterize both tumor cells and the surrounding microenvironment in diverse tissue types, as well as features that enable the selection of regions of interest down to a few or even single cells.

## Conclusions

Collectively, the advances presented at AGBT highlighted exciting technical developments in de novo genome assembly, single-cell sequencing, and spatially resolved profiling. Many presentations at the conference impressed with an array of creative applications of these methods to address a range of biological problems from organismal development to metagenomics, ecology, and conservation. We anticipate hearing of additional applications for many of these new technologies at next year’s meeting, including in the clinical genomics space, as well as an increasing focus on single-cell and spatially resolved measurements.

## Abbreviations

AGBT: Advances in Genome Biology and Technology
ATAC: Assay for transposase-accessible chromatin
CITE-Seq: Cellular indexing of transcriptomes and epitopes by sequencing
CNV: Copy number variant/variation
DCIS: Ductal carcinoma in situ
DNA: Deoxyribonucleic acid
FFPE: Formalin-fixed paraffin-embedded
GESTALT: Genome editing of synthetic target arrays for lineage tracing
GWAS: Genome-wide association study
Hi-C: High-throughput chromosome conformation capture
IDC: Invasive ductal carcinoma
mRNA: Messenger ribonucleic acid
PBMC: Peripheral blood mononuclear cell
RNA: Ribonucleic acid
RNA-seq: RNA sequencing
SNV: Single nucleotide variant/variation
SV: Structural variant/variation
TSCS: Topographical single cell sequencing
UV: Ultraviolet

